# Radiation-Induced Baroreflex Dysfunction: A Rare Case of Severe Orthostatic Hypotension

**DOI:** 10.7759/cureus.94423

**Published:** 2025-10-12

**Authors:** Paolo Nikolai So, Abdul-Rahaman Adedolapo Ottun, Saint-Martin Allihien, Saheed Soleye, Julio Pena

**Affiliations:** 1 Internal Medicine, Piedmont Athens Regional Medical Center, Athens, USA; 2 Internal Medicine, Texas A&amp;M University Naresh K. Vashisht College of Medicine, San Angelo, USA; 3 Nephrology, Georgia Kidney Consultants, Watkinsville, USA

**Keywords:** afferent baroreflex failure, autonomic failure, baroreflex dysfunction, baroreflex failure, efferent baroreflex failure, orthostatic hypotension, radiotherapy complications

## Abstract

Baroreflex failure is an under-recognized and often overlooked cause of orthostatic hypotension, particularly following head and neck radiation therapy. We present an 88-year-old man who developed recurrent falls and a distinctive pattern of supine hypertension with systolic pressures reaching 235 mmHg and orthostatic hypotension as low as 66/44 mmHg three weeks after completing treatment for basal cell carcinoma. A comprehensive workup, including brain imaging, telemetry, serum catecholamines, thyroid function, and immunofixation electrophoresis, excluded structural, endocrine, and infiltrative causes of autonomic dysfunction. Radiation-induced baroreflex failure was inferred clinically based on pronounced positional blood pressure (BP) variability, lack of reciprocal heart rate changes, and the temporal relationship to recent therapy. Treatment with droxidopa, compression therapy, and a carefully titrated antihypertensive regimen led to improvement in orthostatic tolerance. Titration prioritized relief of symptomatic hypotension with droxidopa before addressing nocturnal hypertension, which was managed non-reactively using low-dose amlodipine. The patient remained stable throughout the 10-month follow-up with sustained symptom improvement. This case underscores the importance of recognizing radiation as a potential cause of baroreflex failure in patients with significant polyvascular disease, expanding the spectrum of presentations to include earlier onset than historically reported.

## Introduction

Orthostatic hypotension (OH) is defined as a sustained decrease in blood pressure (BP) of at least 20 mmHg systolic or 10 mmHg diastolic within three minutes of standing upright or during a greater than 60-degree head-up tilt [1]. It is a common condition among older adults, affecting approximately 20% of individuals over 60 years living independently and nearly 25% of elderly individuals in long-term care facilities [2]. It is associated with adverse outcomes, including syncope, falls, and injury, and is linked to increased cardiovascular morbidity and mortality [3,4]. Data from the Nationwide Inpatient Sample revealed a marked age-related rise in OH hospitalizations in the United States, with rates reaching 233 admissions per 100,000 individuals aged 75 or older, with average hospital stays lasting three days and in-hospital mortality of 0.9% [5].

Several factors contribute to OH development, including medications, comorbidities such as heart failure and diabetes, acute illnesses, dehydration, and underlying autonomic dysfunction [6]. Distinguishing neurogenic OH secondary to impaired baroreflex-mediated sympathetic activation from non-neurogenic causes such as hypovolemia or medication effects is important for guiding therapy.

The baroreflex maintains cardiovascular stability through sensors in the carotid sinus and aortic arch that transmit signals via cranial nerves IX and X to the medulla, which adjusts sympathetic and parasympathetic output. Disruption anywhere along this pathway can cause baroreflex failure, a rare but important cause of refractory OH. Baroreflex failure classically presents with alternating severe supine hypertension and profound OH, often with BP differences exceeding 100 mmHg between positions [7]. Acute forms usually follow bilateral carotid surgery or neck trauma, while radiation-induced cases tend to manifest later, often years after treatment [7-10].

Neck radiation can cause early impairment of baroreflex function, but overt failure develops in only a minority of patients and usually after progressive fibrosis of the carotid sinus [9]. No clear cutoff separates acute from late effects, but exposure time, inflammatory markers (e.g., high-sensitivity C-reactive protein), and carotid intima-media thickness have been proposed as predictors of post-radiation autonomic dysfunction [11]. Although delayed manifestations are more common, our case illustrates refractory OH secondary to radiation-induced baroreflex failure, presenting more acutely in the setting of predisposing factors such as diabetes and bilateral carotid artery stenosis.

## Case presentation

An 88-year-old male with polyvascular disease (coronary artery disease status post bypass, bilateral carotid artery stenosis, peripheral vascular disease, type 2 diabetes mellitus, vascular dementia), chronic diastolic heart failure, hypertension, and chronic kidney disease stage 3 presented to the emergency department after a witnessed fall without loss of consciousness. He sustained superficial abrasions on his right forearm. He denied dizziness, nausea, vomiting, or diarrhea. He experienced no sudden feeling of warmth or visual dimming before the fall. He had no new medications, vices, or identifiable stressors. Three weeks prior to the presentation, he had completed radiation therapy for basal cell carcinoma of the head and neck. Details of the carcinoma report and the exact irradiation field were not available for review. He had been asymptomatic prior to radiotherapy.

On admission, the patient appeared euvolemic. Orthostatic vitals revealed a supine BP of 179/76 mmHg, dropping to 76/38 mmHg on standing. This pattern worsened during his stay, with supine pressures exceeding 200 mmHg to 230 mmHg and standing readings persistently low (60s-90s systolic) (Table 1). Heart rate responses were minimal (e.g., HR 66 supine to 77 standing), suggesting impaired baroreflex function.

**Table 1:** Blood pressure and heart rate measurements during hospitalization

Day of admission	Supine BP (mmHg)	Heart rate (bpm)	Sitting BP (mmHg)	Heart rate (bpm)	Standing BP (mmHg)	Heart rate (bpm)
1	179/76	66	140/65	70	76/38	77
2	224/99	84	168/80	—	92/52	—
3	186/77	—	96/51	59	—	—
4	223/103	81	98/52	—	—	—
5	229/105	91	164/76	97	66/44	97
6	198/91	67	148/78	67	83/49	59
8	235/105	68	198/88	—	96/55	—
9	222/98	66	88/51	82	—	—
11	209/93	—	103/55	—	—	—
13	177/77	—	129/58	—	94/53	—
14	204/84	—	88/50	—	—	—
19	129/60	—	84/51	—	—	—

**Figure 1:**
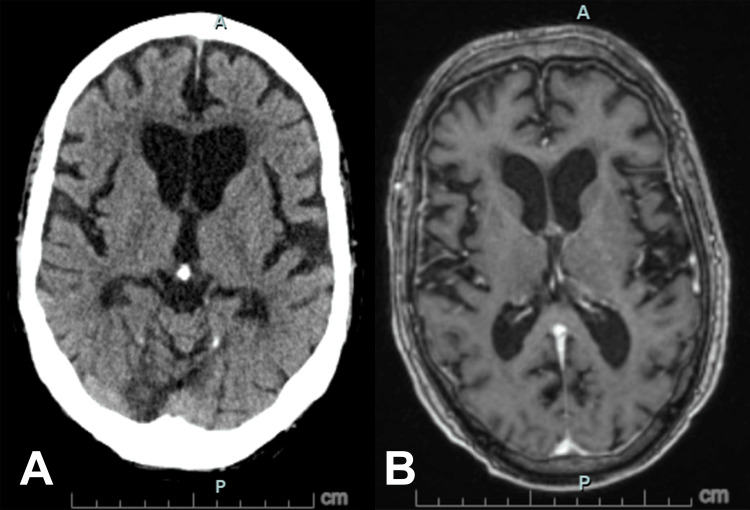
Axial non-contrast CT (A) and post-contrast T1-weighted MRI (B) of the brain showing no acute intracranial abnormality, with age-related changes noted.

Imaging from a head CT scan and brain MRI with and without contrast excluded stroke and normal pressure hydrocephalus (Figure 1). Carotid imaging was not performed, given his known bilateral carotid artery stenosis. A 12-lead electrocardiogram and subsequent telemetry monitoring showed no cardiac arrhythmias. Laboratory evaluation showed normal findings, except for mild hyperglycemia (Table 2). Catecholamine levels were within normal limits, while serum immunofixation electrophoresis excluded amyloidosis.

**Table 2:** Laboratory Findings AST: Aspartate aminotransferase, ALT: Alanine aminotransferase, TSH: Thyroid-stimulating hormone

Parameter	Result	Reference range
White blood cells (×10³/μL)	9.70	4.0-10.5
Hemoglobin (g/dL)	12.9	13.8-17.2
Platelets (×10³/μL)	212	130-400
Sodium (mmol/L)	134	133-145
Potassium (mmol/L)	4.8	3.3-5.1
Chloride (mmol/L)	101	98-108
CO2 (mmol/L)	25	22-32
Anion gap	13	8-16
Glucose (mg/dL)	269	70-100
Blood urea nitrogen (mg/dL)	18	6-20
Creatinine (mg/dL)	1.32 (baseline 1.23)	0.50-1.20
Calcium (mg/dL)	8.8	8.4-10.2
Albumin (g/dL)	3.6	3.0-5.0
AST (U/L)	24	12-50
ALT (U/L)	17	7-52
Hemoglobin A1c (%)	7.6	4.3-6.1
TSH (mIU/L)	2.086	0.34-5.60
Lactate (mmol/L)	1.90	0.50-2.00
Epinephrine (pg/mL)	40	<58
Norepinephrine (pg/mL)	525	149-564
Dopamine (pg/mL)	42	<16
Serum protein electrophoresis	Isolated albumin decrease	
Serum immunofixation	Normal pattern	
Urine microalbumin creatinine ratio (ug/mg)	536	0-30
Urine protein creatinine ratio	0.99	0.00-0.20

Full autonomic reflex testing was not available at our institution. Instead, a simple bedside diagnostic approach was used: the ratio of heart rate increase (beats per minute) to systolic BP decrease (mmHg) during orthostatic challenge. A value <0.5 supported a neurogenic cause, consistent with baroreflex failure. Overall, based on the alternating pattern of severe supine hypertension and orthostatic hypotension, absent compensatory heart rate responses, systematic exclusion of other causes, and temporal relationship with radiotherapy, radiation-induced baroreflex failure was deemed the most likely diagnosis.

At presentation, the patient's home antihypertensive medications (diltiazem 180 mg daily, losartan-hydrochlorothiazide 100/12.5 mg daily) were withheld to avoid worsening hypotension. Non-pharmacologic measures included compression stockings, abdominal binders, gradual positional changes, and hydration. Initial pharmacologic management with midodrine and fludrocortisone was ineffective and discontinued due to lack of response. Droxidopa was started on day 13 at 100 mg three times daily, resulting in immediate improvement in orthostatic vitals (Table 1). The dose was titrated to 200-200-100 mg. Following droxidopa initiation, amlodipine 10 mg nightly was introduced to control nocturnal hypertension and was later adjusted to 5 mg nightly.

The patient was discharged on hospital day 21 with improved orthostatic tolerance and referred to a specialized autonomic center for ongoing therapy. He and his family were counseled regarding the chronic nature of baroreflex dysfunction and long-term management strategies. After discharge, he also underwent physical therapy. Over a 10-month follow-up, his BP remained stable with sustained clinical improvement, reporting improved mobility and strength. His current regimen includes droxidopa 200 mg three times daily, fludrocortisone 0.1 mg nightly, and amlodipine 5 mg nightly.

## Discussion

The carotid arterial baroreflex plays a vital role in regulating BP and maintaining cardiovascular homeostasis. Stretch receptors in the carotid sinus detect changes in BP and transmit signals via afferent nerves to brainstem nuclei, which in turn modulate sympathetic and parasympathetic activity to maintain hemodynamic stability. Disruption at any point along this reflex arc can result in significant cardiovascular dysregulation, producing distinct yet sometimes overlapping cardiovascular phenotypes [7].

When afferent neurons involving cranial nerves IX and X are damaged, mechanosensory input to the nucleus of the solitary is disrupted, leading to unchecked activity of premotor sympathetic neurons in the rostral ventrolateral medulla [12]. This leads to unopposed sympathetic activity and excessive norepinephrine release, universally manifesting as hypertension [9]. Although clinical manifestations can vary, patients often exhibit labile BP with alternating surges of severe hypertension and episodes of symptomatic hypotension. They could also present with acute hypertensive crises triggered by mental stress, accompanied by tachycardia, headaches, and facial flushing [7,9,10,12]. While orthostatic hypotension can be present, there is often no additional autonomic dysfunction [12].

In contrast, impairment of the efferent arm of the baroreflex results in blunted sympathetic activation. Impaired norepinephrine release at the neurovascular junction limits vasoconstriction, causing OH and symptoms of organ hypoperfusion. Approximately half of these patients develop supine hypertension due to residual sympathetic activity and vascular hypersensitivity, with loss of the normal nocturnal BP dip [12]. Other autonomic fibers are also frequently affected, including bladder, gastrointestinal, and sexual functions. This broader autonomic involvement supports the classification of efferent baroreflex failure alongside central autonomic disorders under the collective term of autonomic failure [10,12].

Our patient demonstrated features of both afferent and efferent failure: severe orthostatic hypotension with inadequate heart rate response and intermittent supine hypertension without other autonomic organ involvement, often seen in afferent failure. Full autonomic reflex testing (Valsalva, tilt-table, quantitative sudomotor testing) was unavailable, but a bedside ratio of heart rate increase to systolic BP fall (0.1; Table 1) confirmed a neurogenic origin. This favored baroreflex failure over non-neurogenic causes [12]. His diabetes mellitus likely contributed to efferent failure, while radiation exposure implicated afferent injury. Afferent baroreflex failure has been reported as a complication of head and neck irradiation [7,10-14]. However, injury to cranial nerves carrying both afferent and efferent fibers, such as the vagus nerve, may also contribute, producing varied clinical presentations depending on the extent of collateral damage to efferent pathways [10]. In addition, although vascular dementia inherently carries a risk of autonomic dysfunction [15], other neurodegenerative causes such as synucleinopathies could not be entirely excluded.

A notable feature of this case is the abrupt onset of symptoms following the initiation of radiotherapy. Although typically a delayed consequence due to progressive fibrosis and injury to the carotid sinus, measurable baroreflex impairment can occur soon after radiation exposure [9]. While diabetes and bilateral carotid disease can independently impair baroreflex function, the temporal proximity to radiotherapy, the characteristic hemodynamic pattern, and exclusion of other causes strongly support radiation as the precipitating factor. In this patient, preexisting polyvascular disease likely lowered the threshold for decompensation, leading to earlier and more severe manifestation of baroreflex failure. This presentation may also reflect the unmasking of subclinical dysfunction in the context of comorbidities. Overall, this case broadens the spectrum of reported presentations by showing that an acute onset can occur in patients with significant vascular disease.

Management of baroreflex dysfunction is challenging due to a scarcity of evidence-based treatment guidelines [9]. Patients and caregivers should be counseled that normalization of BP is rarely achievable; instead, therapy aims to reduce symptom burden and prevent complications. Treatment strategies also differ between afferent and efferent failure. Afferent lesions are more often managed with central sympatholytics to blunt hypertensive surges, while efferent failure relies on pressor agents to address orthostatic hypotension. In mixed presentations, therapy must be highly individualized. Non-pharmacologic strategies include stress avoidance, adequate hydration, liberal dietary sodium, physical counterpressure maneuvers, and orthostatic training [12]. Pharmacologic intervention requires careful balancing, as OH and supine hypertension frequently coexist and respond oppositely to treatment.

Initial management of OH typically emphasizes non-pharmacologic strategies, including oral water boluses, physical stimulation, and abdominal binders. If medication is required, vasoconstrictors such as midodrine or droxidopa may be used. Fludrocortisone may also be employed to promote intravascular volume expansion [9,12]. In this case, midodrine and fludrocortisone were ineffective, whereas droxidopa, a norepinephrine precursor, produced immediate improvement in orthostatic tolerance.

Conversely, hypertensive episodes should not be managed reactively with short-acting antihypertensives, as it may exacerbate BP volatility. Instead, long-acting central sympatholytic agents (methyldopa, guanfacine, or transdermal clonidine) are preferred. These should be administered at the lowest effective dose necessary to blunt sympathetic surges while minimizing risk of hypotension. Adjunctive antihypertensives should be selected based on comorbid conditions [9]. In our patient, long-acting amlodipine at night provided stable control.

Long-term outcomes in radiation-induced baroreflex failure remain poorly defined due to the limited number of reported cases. However, OH in diabetes mellitus was associated with a higher risk of total mortality and cardiovascular events [16]. In this case, stability and functional improvement were sustained over 10 months of follow-up, suggesting that durable benefit is possible with appropriate therapy.

## Conclusions

Overall, our case highlights the importance of recognizing radiation-induced baroreflex dysfunction as a rare but clinically significant cause of orthostatic hypotension. It may accelerate or unmask subclinical dysfunction in those with preexisting significant comorbidities such as diabetes and bilateral carotid artery disease, leading to earlier clinical manifestations than what is historically reported. Prompt recognition is essential to guide therapy. Optimal management requires a highly individualized, multimodal approach tailored to the underlying pathophysiology, balancing orthostatic tolerance with the risks of supine hypertension.

## References

[1] Freeman R, Wieling W, Axelrod FB (2011). Consensus statement on the definition of orthostatic hypotension, neurally mediated syncope and the postural tachycardia syndrome. Clin Auton Res.

[2] Saedon NI, Pin Tan M, Frith J (2020). The prevalence of orthostatic hypotension: a systematic review and meta-analysis. J Gerontol A Biol Sci Med Sci.

[3] Eigenbrodt ML, Rose KM, Couper DJ, Arnett DK, Smith R, Jones D (2000). Orthostatic hypotension as a risk factor for stroke: the atherosclerosis risk in communities (ARIC) study, 1987-1996. Stroke.

[4] Rose KM, Tyroler HA, Nardo CJ (2000). Orthostatic hypotension and the incidence of coronary heart disease: the Atherosclerosis Risk in Communities study. Am J Hypertens.

[5] Shibao C, Grijalva CG, Raj SR, Biaggioni I, Griffin MR (2007). Orthostatic hypotension-related hospitalizations in the United States. Am J Med.

[6] Sathyapalan T, Aye MM, Atkin SL (2011). Postural hypotension. BMJ.

[7] Biaggioni I, Shibao CA, Jordan J (2022). Evaluation and diagnosis of afferent baroreflex failure. Hypertension.

[8] Sharabi Y, Dendi R, Holmes C, Goldstein DS (2003). Baroreflex failure as a late sequela of neck irradiation. Hypertension.

[9] Biaggioni I, Shibao CA, Diedrich A, Muldowney JA 3rd, Laffer CL, Jordan J (2019). Blood pressure management in afferent baroreflex failure: JACC review topic of the week. J Am Coll Cardiol.

[10] Ketch T, Biaggioni I, Robertson R, Robertson D (2002). Four faces of baroreflex failure: hypertensive crisis, volatile hypertension, orthostatic tachycardia, and malignant vagotonia. Circulation.

[11] Huang CC, Huang TL, Hsu HC (2013). Long-term effects of neck irradiation on cardiovascular autonomic function: a study in nasopharyngeal carcinoma patients after radiotherapy. Muscle Nerve.

[12] Kaufmann H, Norcliffe-Kaufmann L, Palma JA (2020). Baroreflex dysfunction. N Engl J Med.

[13] Beishon LC, Hosford P, Gurung D (2022). The role of the autonomic nervous system in cerebral blood flow regulation in dementia: a review. Auton Neurosci.

[14] Farach A, Fernando R, Bhattacharjee M, Fuentes F (2012). Baroreflex failure following radiotherapy for head and neck cancer: a case study. Pract Radiat Oncol.

[15] Goodman BP, Schrader SL (2009). Radiation-induced cranial neuropathies manifesting as baroreflex failure and progressive bulbar impairment. Neurologist.

[16] Zhou Y, Ke SJ, Qiu XP, Liu LB (2017). Prevalence, risk factors, and prognosis of orthostatic hypotension in diabetic patients: a systematic review and meta-analysis. Medicine (Baltimore).

